# Obesity Causes Abrupt Changes in the Testicular Microbiota and Sperm Motility of Zebrafish

**DOI:** 10.3389/fimmu.2021.639239

**Published:** 2021-06-25

**Authors:** Yufang Su, Liting He, Zhiyong Hu, Ying Li, Yuan Zhang, Zunpan Fan, Kai Zhao, Huiping Zhang, Chunyan Liu

**Affiliations:** ^1^ Institute of Reproductive Health, Tongji Medical College, HuaZhong University of Science and Technology, Wuhan, China; ^2^ Department of Oncology, Jiangxi Maternal and Child Health Hospital, Nanchang, China; ^3^ Prenatal Diagnostic Center, People’s Hospital of Guangxi Zhuang Autonomous Region, Nanning, China

**Keywords:** testicular microbiota, obesity, zebrafish, intestinal microbiota, sperm motility

## Abstract

**Background:**

Obesity is a recognized risk factor for low fertility and is becoming increasingly prevalent in many countries around the world. Obesity changes intestinal microbiota composition, causes inflammation of various organs, and also reduces sperm quality. Several microorganisms are present in the testis. However, whether obesity affects the changes of testicular microbiota and whether these changes are related to reduced fertility in obese men remain to be elucidated.

**Methods:**

In the present study, a zebrafish obesity model was established by feeding with egg yolk powder. Sperm motility was measured by the Computer Assisted Sperm Analysis system, testicular microbial communities was assessed *via* 16s RNA sequencing, the immune response in zebrafish testis was quantified by quantitative real-time polymerase chain reaction and enzyme-linked immunosorbent assay, and the testicular tissue structure was detected by electron microscopy and hematoxylin–eosin staining.

**Results:**

Compared with the control group, zebrafish sperm motility was dramatically reduced, the expression of testicular proinflammatory cytokines in the testes was upregulated, and the blood–testis barrier structure was disrupted in the obese group. In addition, testicular microbiome composition was clearly altered in the obese group.

**Conclusion:**

Obesity alters testicular microbiota composition, and the reason behind the decreased sperm motility in obese zebrafish may be related to changes in the testicular microbial communities.

**Graphical Abstract:**
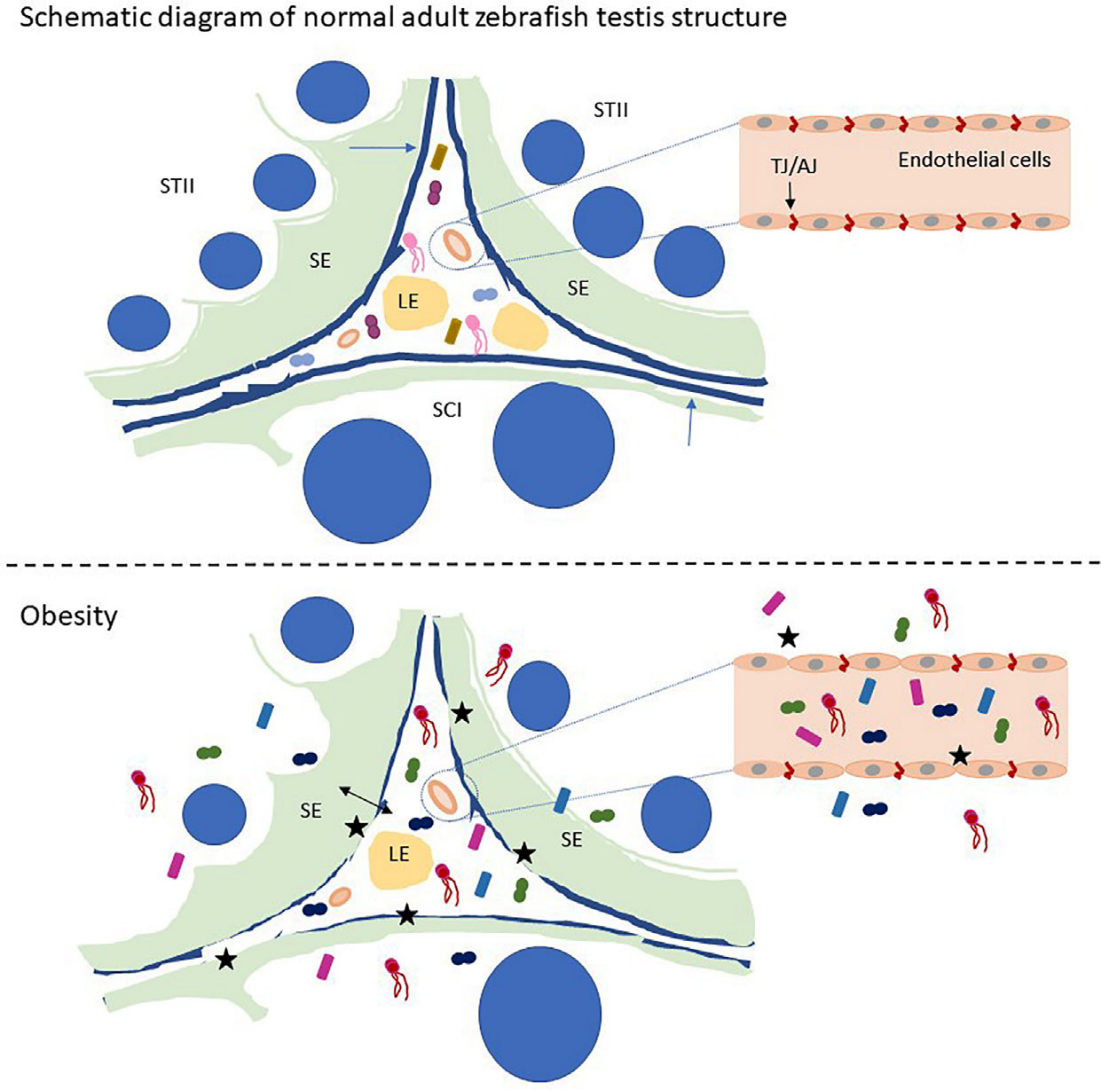
Schematic of zebrafish testis microbiological disturbance and BTB disruption caused by obesity. STII, spermatids at the second phase of spermiogenesis; SCI, primary spermatocytes; LE, Leydig cells in the interstitium; SE, Sertoli cells. The blue arrow indicates the basement membrane of the zebrafish testis, and the black stars indicate the breach of the zebrafish BTB and the vascular barrier. Small graphics in the picture represent microorganisms.

## Highlights

Changes in the composition of testicular microbiota in obese zebrafish.

Decreased sperm motility in obese zebrafish.
Defective tight junction of blood–testis barrier in obese zebrafish.
Zebrafish testicular immune response is activated.
Altered testicular microbiota in obese zebrafish are related to changes in their gut microbes.

## Introduction

In recent decades, the number of obese men of childbearing age has almost doubled (1, 2). In 2000, 65% of adult men had a body mass index (BMI) over 25 (overweight), and 30% had a BMI higher than 30 (obesity) (3). Cohort analysis demonstrated that the total sperm count and sperm motility were lower in obese than in healthy donors (4, 5). Endocrine disorders (disorders of testosterone levels) (6, 7), genetics, autophagy (8), and physical or chemical factors are all involved in low fertility caused by obesity in men.

There are approximately 10^13^–10^14^ bacteria in the human intestine (9). The gut microbiota can regulate liver metabolism by reducing energy expenditure and promoting obesity (10). *Faecalibacterium prausnitzii*, an anti-inflammatory bacterium, was found to be significantly decreased in the intestine of morbidly obese diabetic patients (11, 12). In previous studies, obese mice fed on a high-fat diet (HFD) had increased intestinal permeability, and the abundance of *Bacteroides*, *Clostridium*, and *Bifidobacterium* in the intestine of mice decreased by 50% (13, 14). The richness of *Enterobacter cloacae strain B29* was significantly aggrandized in the intestinal microorganisms of obese individuals (15, 16). The above findings demonstrated that High-fat-diet reduces the abundance of predominant bacteria in the intestine and accelerates the richness of pathogenic bacteria, thereafter the metabolites of the changed microbiota may contribute to obesity, which destroys the intestinal vascular barrier. The human testis has tissue-associated symbiotic bacteria, with *Actinomycetes*, *Bacteroides*, *Pachybacteria*, and *Proteus* being the most abundant microorganisms in the men with testicular tumor (seminoma) with normozoospermic (17). A study showed that *Clostridium* spp. are related to the vitality and morphology of human sperm (18). Cohort studies have found that an increase in *Actinomycetes* and *Sclerotinia* changes the testicular microbiota in male patients with non-obstructive azoospermia. Moreover, patients with complete germ cell aplasia did not have Clostridia in their testes (17). All of the above findings suggest that changes in testicular microorganisms may be associated with decreased male fertility.

An article found that adult males over 35 years of age may develop epididymitis caused by intestinal pathogens (19). Studies have shown that the permeability of the blood–testis barrier (BTB) is increased and testicular development is altered in obese mice when their intestinal microbiota composition was changed (20–22). However, whether obesity changes the testicular microorganism composition and whether defects in testicular function caused by obesity are related to changes in testicular microorganism composition remain to be elucidated. Thus, we constructed a zebrafish obesity model through HFD feeding. After 8 weeks, the sperm motility and integrity of the BTB were measured. Based on 16s RNA sequencing, the testicular and intestinal microbial community composition was analyzed to reveal the association between obese infertility and testicular microbiota.

## Materials and Methods

### Chemicals and Materials

Egg yolk powder (59% fat, 32% proteins, 2% carbohydrates) with a purity >98% was obtained from Solarbio (Cat # E8200, China). Fragments and coverslip for zebrafish sperm motility testing were purchased from Hamilton Thorne - 203L-72 (Lot # 559599, USA). Oil Red O was purchased from Sigma (Lot # SLBT6544, USA).

### Fish Maintenance and HFD Feeding

Zebrafish embryos were provided by the Institute of Reproductive Health, Tongji Medical College, Huazhong University of Science and Technology. Zebrafish were maintained in a flow-through system containing charcoal-filtered water on a 14 h light/10h dark photoperiod at 28 ± 0.5°C.

AB zebrafish strains were utilized because these strains have become the most commonly used zebrafish for studying obesity and obesity-related experiments (22–25). We randomly assigned 80 3-month-old adult zebrafish to two diet groups. One group (40 per group) was fed with red worms to maintain physiological energy requirements, whereas the other group was given 30 mg of egg yolk powder per fish per day while feeding an equivalent number of red worms. Zebrafish were maintained in 5 L tanks for every 10 fish and were fed twice daily. They were fasted overnight and sacrificed in the eighth week (26).

### Measurement of Zebrafish Length, Weight, Blood Glucose, and Cholesterol

The body weight and length of zebrafish were recorded and calculated to obtain the BMI. We collected blood samples from the dorsal artery and pooled blood samples for every 8 fish for one exemplary feeding experiment. Fasting blood glucose was measured using a glucometer (Safe blood glucose meter, Sannuo), and cholesterol levels were determined using the Amplex^®^ Red Cholesterol Assay Kit (Invitrogen) (26).

### Histology

Cryosections from zebrafish liver were prepared by embedding freshly isolated liver tissue in 4% paraformaldehyde (Sigma‐Aldrich, St. Louis, Missouri, USA). The slides were stained at room temperature with Oil Red O in the dark overnight, and images were captured under an Olympus microscope (Tokyo, Japan) (27). Anatomically comparable sections of subcutaneous fat were stained with hematoxylin–eosin (HE), and microscopic images were obtained at 40× magnification under an Olympus microscope (Tokyo, Japan). Put a fresh zebrafish testis sample of about 1-2 mm^3^ into the electron microscope fixation solution within 2 minutes, fix it with osmium acid, dehydrate, infiltrate, and embed it, and cut it into a thickness of 80-100nm (Leica, EM UC7, Germany), double staining with uranium and lead, and dry at room temperature Overnight, observe the microstructure of the tissue under the electron microscope (FEI, Tecnai G2 20 TWIN, American).

### Zebrafish Sperm Motility Test

Zebrafish semen was manually squeezed out and placed in a 100 μL Eppendorf tube with 10 μL of D-Hank’s solution. The mixed droplets of sperm were placed on a 20 μm slide and then added with 5 μL of 0.1% bovine serum albumin solution (activated). The slide was immediately covered with a coverslip and pushed under the HT Computer Assisted Sperm Analysis (CASA) II Animal (Hamilton Thorne, USA). Each group randomly selected 6 fish for sperm motility test, and repeated the test for each fish 5 times, and the average was determined. Sperm motility test for each fish was completed within 1 h after fresh zebrafish semen was collected.

### Enzyme-Linked Immunosorbent Assay

Serum IL-1β levels were determined by using the Fish IL-1β ELISA Kit (MyBioSource), and serum testosterone levels were quantified by using Testosterone ELISA Kit (Cayman) following the manufacturer’s instructions.

### Detection of Gene Expression

Prepare enough sterile dissecting instruments, anesthetize the zebrafish on ice, and extract fresh zebrafish testis samples in a sterile ultra-clean bench. Each zebrafish dissection instrument is not reused. DNA was extracted from the fresh testicular tissue according to the instructions of the EZNA^®^ Soil DNA Kit (Omega Biotechnology Company, Norcross, Georgia, USA). We pooled the bilateral testes of 10 zebrafish in each group into an experimental group for DNA extraction. Total RNA was extracted from zebrafish testis by using TRIzol reagent (Takara Biochemicals, Japan) following the manufacturer’s protocol. RNA reverse transcriptase reaction was conducted using a PrimeScript RT kit (Takara, Kusatsu, Japan). Real-time polymerase chain reaction (RT-PCR) was performed on a StepOnePlus Real-Time PCR instrument (Applied Biosystems). The gene expression levels of *β-actin*, *tnf-α*, *il-1β*, and *il-8* were detected *via* an SYBR Green system (DBI Bioscience) using oligonucleotide primers (**Table 1**) (28, 29). Each tested gene was repeated three times for qRT-PCR.

**Table 1:** Sequences of primer pairs used in the real-time quantitative PCR reactions.

Gene	Primer sequences (from 5’ to 3’)	Size (bp)
*β*-*actin*	F: ATGGATGAGGAAATCGCTGCC	127
	R: CTCCCTGATGTCTGGGTCGTC	
*tnf*-*α*	F: GGGCAATCAACAAGATGGAAG	250
	R: GCAGCTGATGTGCAAAGACAC	
*il*-*1β*	F: TGGTGGATTCAGTGCCGTCT	246
	R: AGGCCAGGTACAGGTTACTTTTG	
*il*-*8*	F: GTCGCTGCATTGAAACAGAA	158
	R: CTTAACCCATGGAGCAGAGG	

### 16s RNA Sequencing and Bioinformatics Analysis

Taking into account that zebrafish are aquatic animals, feces are not easy to collect, our gut microbes and testis microbe specimens come from the entire zebrafish intestine or testis (30). Prepare enough sterile dissecting instruments, anesthetize the zebrafish on ice, and extract fresh zebrafish testis samples in a sterile ultra-clean bench. Each zebrafish dissection instrument is not reused. We pooled the bilateral testes of 10 zebrafish in each group into an experimental group for DNA extraction. DNA was extracted from the fresh testicular tissue according to the instructions of the EZNA^®^ Soil DNA Kit (Omega Biotechnology Company, Norcross, Georgia, USA), and the quality of DNA was detected by 2% agarose gel electrophoresis. The DNA concentration and purity were determined by using NanoDrop2000. Then, the 16S rRNA V3-V4 gene (338F 5’-ACTCCTACGGGAGGCAGCAG-3’ and 806R 5’-GGACTACHVGGGTWTCTAAT-3’) was amplified by PCR, and the PCR product was recovered on a 2% agarose gel. The recovered product was purified by using the AxyPrep DNA Gel Extraction Kit (Axygen Biosciences, Union City, CA, USA) and detected by 2% agarose gel electrophoresis using a Quantus™ Fluorometer (Promega, USA). The NEXTFLEX Rapid DNA-Seq Kit was used to build the library. Sequencing was performed on the Miseq PE300 platform (Illumina).

Trimmomatic software was used for sequencing the original sequences for quality control. FLASH software was employed for splicing. UPARSE software (version 7.1) was applied to cluster the sequences into OTUs based on 97% similarity and remove the chimeras. RDP classifier (2.11) was applied to annotate species classification for each sequence. 16S rRNA sequencing data were analyzed using QIIME 1.9.1 (31). To minimize the effects of false sequences, we deleted OTUs that were less than 0.005% of the total number of sequences and performed data flattening. The sequences were compared using Mothur 1.30.2 for alpha diversity analyses. The genome of the gut microbiome was deduced from the 16S rRNA sequence by PICRUSt (32).

### Data Analyses

Data were quantified as the difference relative to that of the control group and are shown as mean ± standard error of the mean. The data were verified for normality and homogeneity of variance using the Kolmogorov–Smirnov one-sample test and Levene’s test. Intergroup differences were assessed by one-way ANOVA followed by Dunnett’s *post hoc* test. All statistical analyses were conducted by SPSS 18.0. The level of statistical significance was set at *P* < 0.05, indicated by an asterisk.

## Results

### Construction of a Zebrafish Obesity Model

**Figure 1:**
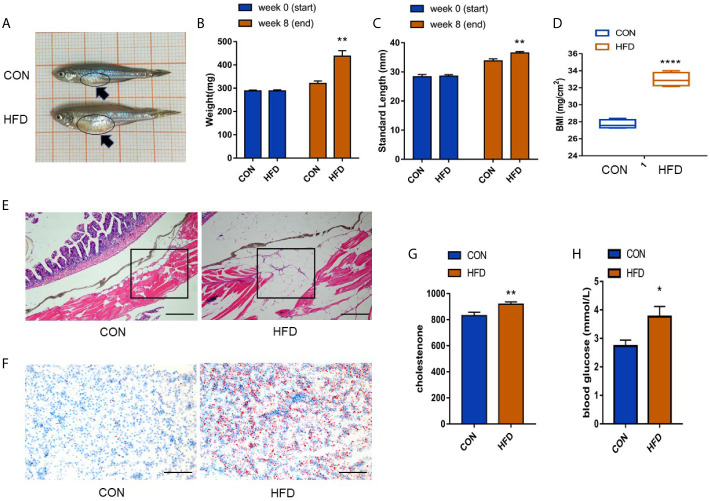
Successful establishment of the obesity model. **(A)** Male zebrafish appearance in control and obese groups. A clearly larger abdomen can be seen in zebrafish in the obese group as shown by the dark blue arrow. **(B, C)** Measurement of body weight and length of zebrafish before and after exposure. **(D)** Significant differences in the BMI index. **(E)** Expression of subcutaneous fat in zebrafish (scale bar = 100 μm). **(F)** Expression of fat droplets in the liver (scale bar = 100 μm). **(G, H)** Determination of cholesterol in the blood and detection of blood glucose. Values are mean ± SME (n = 5). The asterisk represents a statistically significant difference when compared with the controls; *, ** and **** at *P* < 0.05, *P* < 0.01 and *P* < 0.0001, respectively.

After 8 weeks of HFD feeding, we compared parameters related to obesity, including body weight, BMI, and condition index. The presence of early obesity-related metabolic alterations was investigated by quantifying blood glucose levels and cholesterol levels to determine whether the modeling was successful. Compared with the control group, the abdomen of zebrafish in the obese group was enlarged, as shown by the black arrow in **Figure 1A**. The bodyweight increased by approximately 1.4 times, the body length increased by approximately 1.2 times, and the BMI increased from 27–28 to 32–34 (*P* < 0.0001) (**Figures 1B–D**). A significant enhancement in the number of subcutaneous adipocytes in zebrafish was observed. The space between subcutaneous muscle fibers was enlarged by adipocytes, and the volume of each adipocyte increased by approximately 2–3 times under a 10× microscope (**Figure 1E**). Consistent with the expected results, the number of zebrafish liver fat vacuoles was higher in the obese group than in the control group (**Figure 1F**). We detected the expression levels of cholesterol and glucose in the blood of zebrafish, compared with the control group, the blood cholesterol level in the obese group increased (*P* < 0.01) (**Figure 1G**), and the blood glucose levels increased (*P* < 0.05) (**Figure 1H**). The above data indicated the successful construction of the zebrafish obesity model.

### Sperm Motility Decline in the Obese Zebrafish Model

**Figure 2:**
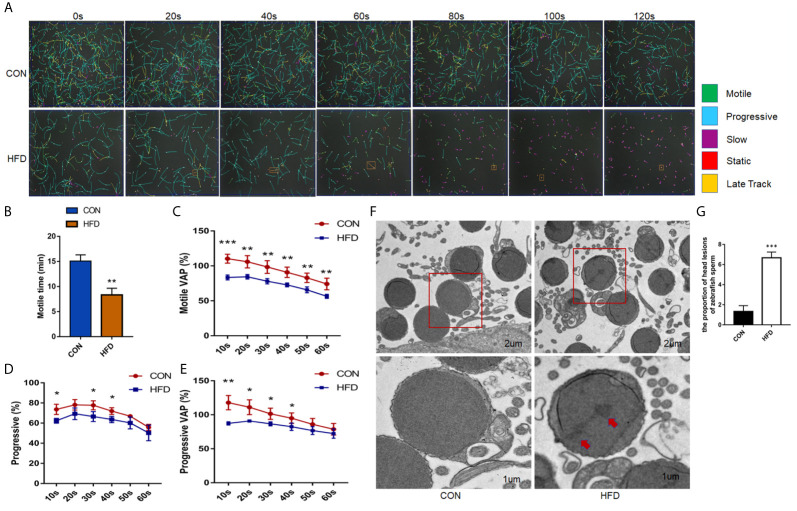
Effects of obesity on the sperm motility of zebrafish. **(A)** Intuitive CASA image of zebrafish sperm movement under a microscope. **(B)** Sperm MOT between the two groups. **(C–E)** Percentage of motile VAP and progressive VAP (%) of adult zebrafish. **(F)** Comparison of sperm morphology between the two groups under the electron microscope. **(G)** The obese group had lesions on the head of the sperm as shown by the red arrow. Data represent mean ± SME (n = 6). *, ** and *** at *P* < 0.05, *P* < 0.01 and *P* < 0.001, respectively.

We analyzed the effects of obesity on zebrafish sperm motility. **Figure 2A** shows a visual representation of zebrafish sperm motility within 120 s, where green indicates motile sperm, blue indicates progressive sperm, purple indicates slow sperm, and red indicates immobile sperm. In 80 s, the number of stationary sperm was higher than that of the control group. The zebrafish motile time and the motile average path velocity (VAP) in the obese group markedly decreased (*P* < 0.01) (**Figures 2B, C**). In addition, the percentage of zebrafish sperm forward motion and the progressive VAP of zebrafish sperm were distinctly reduced (**Figures 2D, E**). Electron microscopy revealed that the zebrafish sperm heads in the obese group had lesions, as shown by the red arrow in **Figure 2F**, and the count of head lesions of zebrafish sperm in the obese group was obviously increased in the control group (*P* < 0.001) (**Figure 2G**). The above results indicate that diet-induced obesity reduces the sperm quality of adult zebrafish, which in turn has a negative impact on the fertility of male zebrafish.

### Obesity Destroys Zebrafish BTB Structure and Accelerates Testicular Inflammation

**Figure 3:**
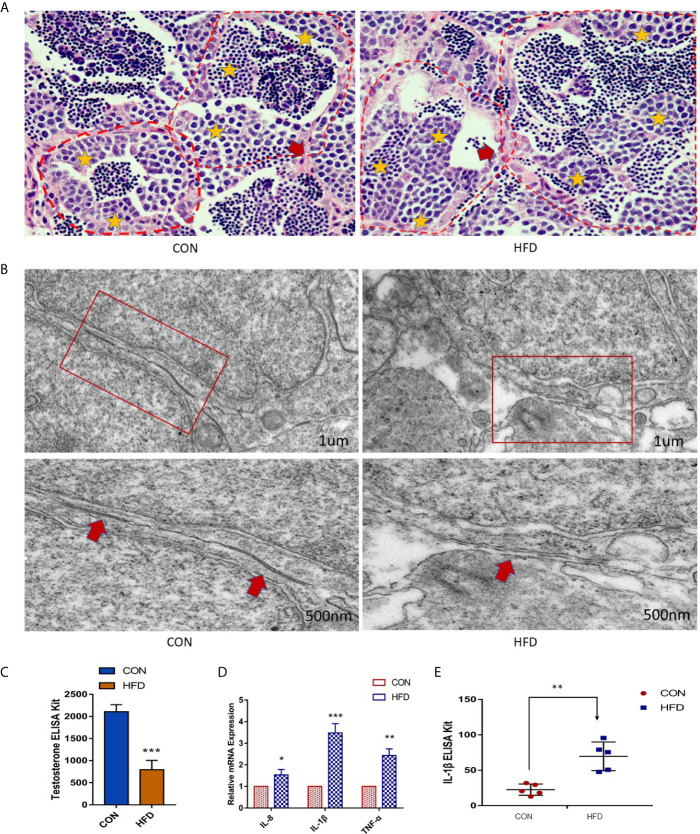
Obesity causes the destruction of zebrafish BTB structure and testicular inflammation. **(A)** Testicular HE staining in the control and obese groups. Yellow stars indicate sperm vesicles of the same type of spermatogenic cells in the zebrafish testis, and the red arrow indicates the interstitial part. Obviously, the zebrafish spermatogenic cells were disorderly arranged, and the interstitium was thickened. **(B)** Ultrastructure of zebrafish BTB. Red boxes indicate connections between Sertoli cells and germ cells in zebrafish testis. In the enlarged image, the BTB structure of the obese group is damaged, as shown by the red arrow. **(C)** Detection of testosterone in the blood. The testosterone level of the obese group was markedly decreased (*P* < 0.001). **(D)** Effects of ODP exposure on the mRNA levels of *tnf-α*, *il-1β*, and *il-8* in zebrafish testis. **(E)** Detection of IL-1β expression in the blood by ELISA. Data represent mean ± SME (n = 5). The asterisk represents a statistically significant difference when compared with the corresponding controls; *, ** and *** at *P* < 0.05, *P* < 0.01 and *P* < 0.001, respectively.

To investigate whether the testicular tissue structure is affected by obesity, we compared the HE staining of zebrafish testes. Unlike mammalian seminiferous tubules, each of the seminiferous vesicles encased by the Sertoli cells in the zebrafish’s seminary is the same type of seminiferous cells. In the **Figure 3A**, as shown by the yellow stars, each seminiferous vesicle contains equally developed spermatogenic cells, and the tissue structure of the seminiferous vesicles and the seminiferous epithelium is clear. The red arrow indicates the area between the two seminiferous epitheliums. However, the testes in the obese group had disordered seminiferous tubules and blurred contour boundaries (**Figure 3A**). We examined the BTB structure under an electron microscope. The control group had a normal BTB physiological structure, whereas the obese group had a significant disorder. Large number of vacuoles, irregular arrangement of tight junction in the gap link between the Sertoli cells and the spermatogenic cells was observed in the obese group (**Figure 3B**). Therefore, obesity increases the permeability of the BTB.

Obesity is related to direct damage to testicular function. Therefore, we investigated the changes in blood testosterone levels between the two groups and found that the plasma testosterone levels in the obese group were dramatically decreased (*P* < 0.001) (**Figure 3C**). Compared with the control group, the expression levels of *il-8*, *tnf-α*, and *il-1β* in zebrafish testes of the obese group were increased (**Figure 3D**). and the protein levels of IL-1β in the plasma of the obese group was increased (*P* < 0.01) (**Figure 3E**). Therefore, our results suggest that diet-induced obesity contributes to the inflammatory response in zebrafish testes.

### High-Fat Diet-Induced Obesity Changes the Intestinal Microorganism Composition

**Figure 4:**
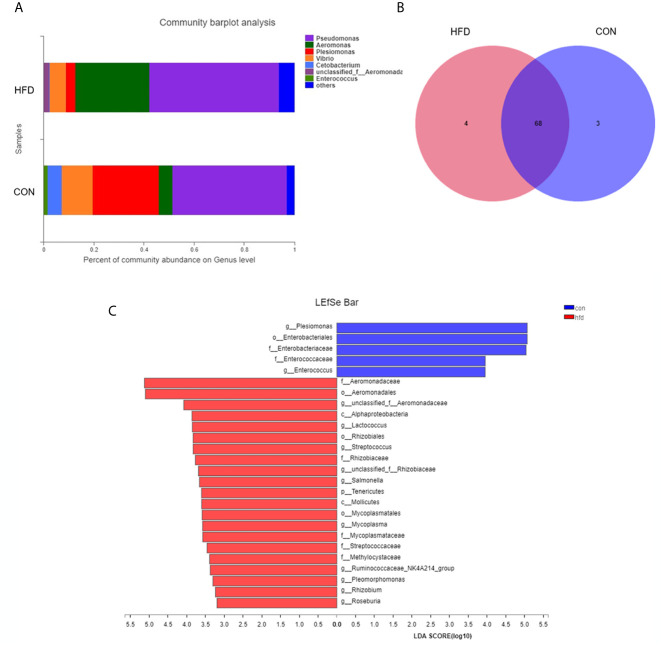
Differences in intestinal microbial composition after High-fat diet-induced obesity. **(A)** Composition of intestinal microbial communities in the control and obese groups. **(B)** Venn plot population analysis results in gut microbes between the control and obese groups. **(C)** LEfSe multi-level species discriminant analysis using non-parametric factorial Kruskal–Wallis rank sum test and LDA to find groups that significantly differ in abundance. Data represent mean ± SME (*n* = 3).

Obesity induced by high-fat diet can change the composition of intestinal microbiota, so we tested the intestinal microbiota in the zebrafish obesity model. Consistent with our expectations, the analysis of the intestinal microbial composition revealed that the abundance of the dominant intestinal bacteria *Plesiomonas* (from 26.39% to 3.87%) and *Vibrio* (from 12.42% to 6.45%) remarkably decreased, and the pathogenic bacteria *Aeromonas* (from 5.35% to 29.49%) dramatically increased in the obese group compared with the control group (**Figure 4A**). At the genus level, the bacterial community compositions in the control and obese groups were also different in terms of abundance and diversity. Each group contained unique bacteria. Sixty-eight types of bacteria and seven species of different bacteria were observed in the two groups (**Figure 4B**). In addition, based on the phylum level abundance indicated that obesity induced by a high-fat diet clearly changes the composition of gut microbes, compared with the control group, *Proteobacteria* (from 97.49% to 91.27%) increased in the obese group, while *Fusobacteria* (from 5.74% to 0.22%) and *Firmicutes* (from 2.38% to 0.97%) decreased (**Figure S1**). Based on LEfSe multi-level species discriminant analysis, we observed a conspicuous difference, in which 26 bacterial groups showed self-evident relative abundance in the obese and control groups (**Figure 4C**), indicating a palpable difference in intestinal microbiota after obesity.

### High-Fat Diet-Induced Obesity Changes the Testicular Microorganism Composition

**Figure 5:**
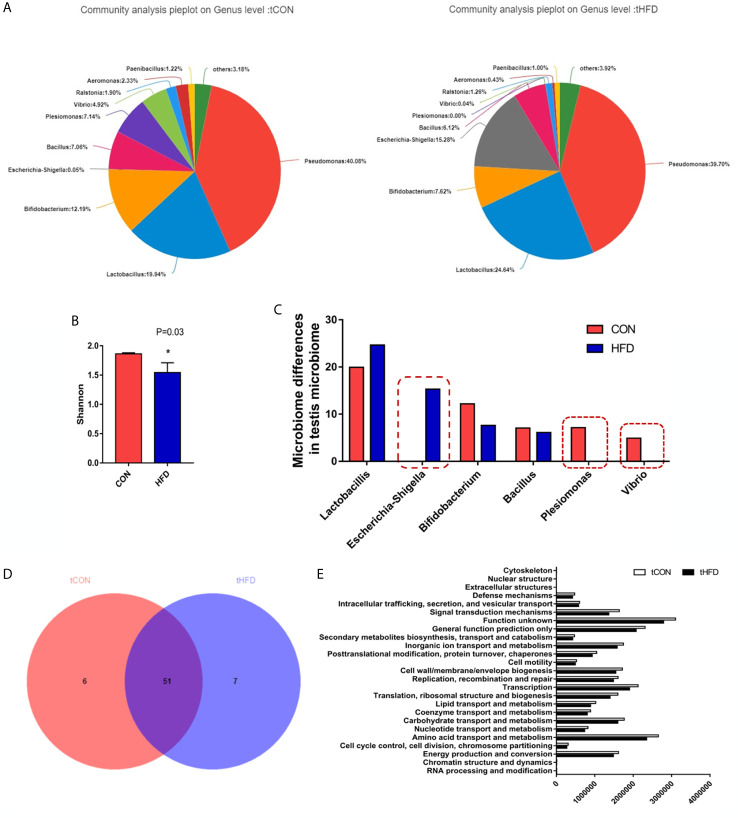
Microbial community composition analysis in the testes of normal and obese male zebrafish showing marked microbial differences between the two groups. **(A)** The pie chart shows the community species composition information of testicular microbes at the gate level in the control and HFD groups. **(B)** Shannon diagram of alpha diversity analysis of testicular microorganisms (*P* = 0.03). **(C)** Analysis and selection of dominant strains of testicular microbial communities that were markedly differently expressed in the two groups. **(D)** Venn plot population analysis results between the two groups. **(E)** Predictive analysis of microbiota function. Data represent mean ± SME (*n* = 3). * at *P* < 0.05.

To understand the relationship between the obesity-related decrease in male fertility and testicular microbial communities, 16s RNA sequencing analysis of the microbial communities was performed. The testis microbe data from one sample of the obese group were excluded because they were deemed non-compliant. The statistical analysis demonstrated that *Pseudomonas*, *Lactobacillus*, and *Bifidobacterium* are the main genera in the testis. However, compared with the control group, the relative abundance of *Lactobacillus* in the obese group was increased, whereas the richness of *Bifidobacterium* decreased (**Figure 5A**). On the phylum level abundance indicated that obesity induced by a high-fat diet changes the testicular microbes, compared with the control group, *Proteobacteria* (from 57.93% to 58.56%) and *Firmicutes* (from 29.61% to 32.84%) increased in the obese group, while and *Actinobacteria* (from 12.39% to 8.36%) decreased (**Figure S2**). Alpha diversity analysis of the testicular microbes revealed that obesity was associated with a reduction in species diversity in the testis (Wilcoxon rank-sum test, *P* = 0.03; **Figure 5B**). Analysis of the differences in the testicular microbiota of the two groups screened out an additional 15.28% of *Escherichia-Shigella* in the obese group. However, *Plesiomonas* and *Vibrio* were not found in the pie chart of the obese group (**Figure 5C**). These data indicated that the diversity of bacteria in the obese group decreased.

In addition, we performed a sample-to-species analysis to display the distribution ratio of dominant species in each group of microbiotas and the distribution ratio of predominant species in different groups (**Figure S3**). At the genus level, each group contained unique bacteria. The bacterial community composition in the control and obese groups was also visibly different in terms of abundance and diversity (**Figure 5D**). Fifty-one common bacterial species were observed in the two groups. The heatmap and sample cluster tree analyses of 50 species of testicular microorganisms in different groups (**Figure S4**) showed significant differences in the predominant testicular microorganism composition after obesity.

To study the changes in the function and metabolism of the microbial community in the testis between the obese and control groups, we deduced the genome from 16S rRNA data and analyzed the functional potential of the intestinal microbiota using PICRUSt. Differences in 25 related genes were screened. In the obese group, the functions of signal transduction mechanism, amino acid transport and metabolism, lipid transport and metabolism, carbohydrate transport metabolism, and coenzyme transport and metabolism all decreased, especially signal transduction mechanism and amino acid transport and metabolism (**Figure 5E**). 16s RNA functional prediction analysis data indicate that the changes in metabolic indications may be related to the decline of sperm motility caused by obesity.

### Comparison Between Testicular Microbiota and Intestinal Microbiota

**Figure 6:**
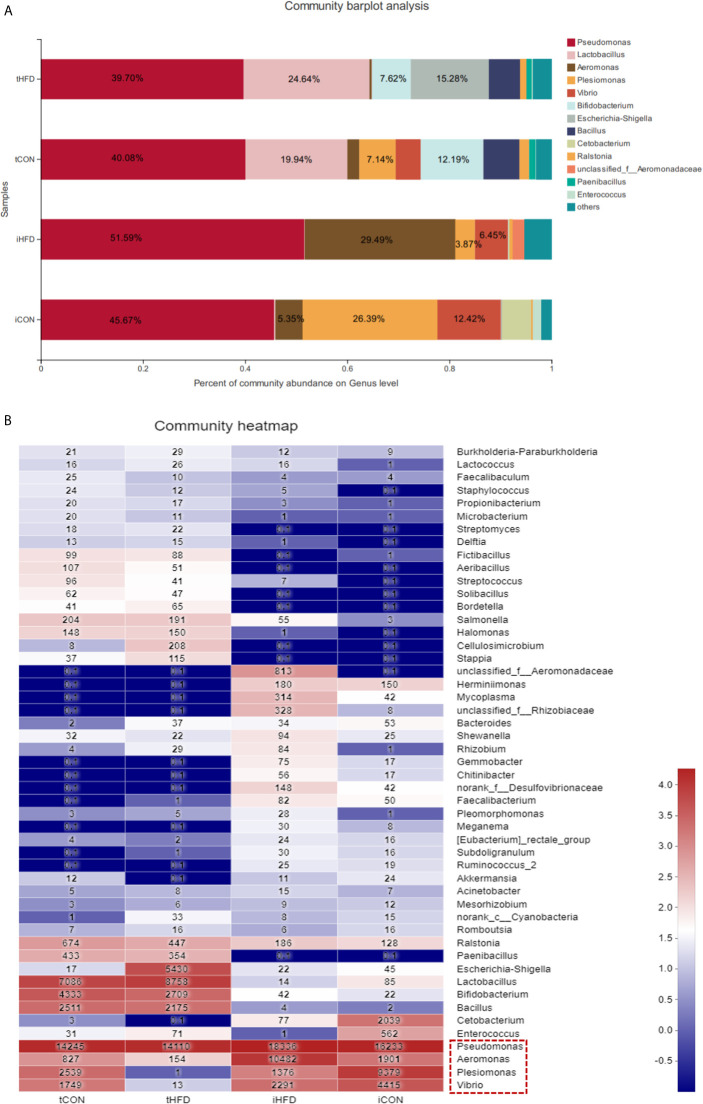
Comparison between testicular and intestinal microorganisms. **(A)** Analysis of the microbial community composition of the testis and intestinal samples in the control and obese groups. The histogram results show that the dominant species are the same at the gate level for different samples, but the relative abundance is different. **(B)** The heatmap shows the species composition and sample cluster tree analysis of 50 species of bacterial communities in the testis and intestinal tracts of different groups at the genus level. Red indicates high bacterial abundance, whereas blue indicates low abundance. Data represent mean ± SME (*n* = 3).

Our results indicated that testicular and intestinal microbes have their predominant microbiota. For instance, *Plesiomona*s (26.39%), *Vibrio* (12.42%), and *Aeromonas* (5.35%) were highly expressed in the control intestinal microorganisms, whereas *Lactobacillus* and *Bifidobacterium* accounted for 19.94% and 12.19% in the testicular microorganisms, respectively. *Pseudomonas* had the highest composition in the gut and testicular microorganisms regardless of whether the zebrafish was obese or not (**Figure 6A** and **Figure S5**). Predominant bacteria such as *Vibrio*, *Plesiomonas*, *Aeromonas*, and *Pseudomonas* were all expressed in normal intestinal and testicular microbes. However, after obesity, the abundance of *Vibrio* and *Plesiomonas* was dramatically decreased in testicular and intestinal microbes (**Figure 6B**). The expression of *Escherichia-Shigella* in the intestinal and testicular microbes of the control group and intestinal microorganisms of the obese group was low (less than 0.15%) but accounted for 15.28% in the testicular microbes of the obese group (**Figures 6A, B**). The above data demonstrate that the composition of predominant intestinal and testicular microorganisms has changed, and there may be a connection between these changes and obesity.

## Discussion

To our knowledge, this study is the first to report on the relationship between obesity and testicular microorganisms. Our results demonstrated that the sperm motility index and blood testosterone levels of the obese group were reduced compared with the control group. Obesity can cause disorders in the BTB structure, and the expression of IL-1β protein remarkably increased in the blood. Based on the above research, we began to consider the role of testicular microbes in obesity and their relationship with intestinal microbes, the changes in testicular microbes, the cause of the changes, and whether these variables may be related to alterations in gut microbes.

Previous studies indicate that the permeability of the testicular BTB was increased in a diet-induced obese mouse model (33). This is consistent with our research results, obesity caused zebrafish disordered seminiferous tubules and blurred contour boundaries, and the structure of the tight junction protein involved in the gap link between the Sertoli cells and the spermatogenic cells was destroyed. BTB permeability may cause testicular inflammation, and cytokines can be employed as markers of inflammation (34, 35). Our results show, the expression levels of *il-8*, *tnf-α*, and *il-1β* in zebrafish testes of the obese group were increased, and the protein levels of IL-1β in the plasma of the obese group was increased in the obese group. Obesity can lead to hypogonadism (lower testosterone levels) in men through the effects of enterotoxin (36), and our results indicated that the zebrafish serum testosterone level decreased after obesity. The above-mentioned obesity leads to the destruction of the zebrafish testis structure, increases the expression of inflammatory factors, and decreases the level of testosterone.

A growing number of evident have shown that obesity is regulated by multiple organs. For instance, certain bacteria and their metabolites may directly target the brain through vagal nerve stimulation or immune nerves, and the endocrine mechanism targets the brain indirectly (37, 38). Chronic kidney disease (39, 40) and non-alcoholic fatty liver disease (41) can cause significant changes in the composition and function of intestinal microbiota, which can cause systemic inflammation. First, our results demonstrated that *Plesiomonas* and *Vibrio* were the predominant testicular microorganisms in the obese group. Moreover, the relative abundance of *Lactobacillus* and *Bifidobacterium* was decreased in the testes of the obese group, indicating that obesity can cause reduced diversity of testicular microorganisms. Moreover, *no rank-c-Cyanobacteria* and *Bacteroides* are normally expressed in the intestinal microbes but not in the testicular microbes of the control group. However, they were observed in the testicular microbes of the obese group. Of note, the abundance of *Escherichia-Shigella* was increased by 15.28% in the obese group, whereas it was less than 0.15% in the control group and intestinal microorganisms. The above findings indicate that there may be a connection between intestinal and testicular microorganisms.

Under healthy conditions, there may be bacterial translocation between adjacent organs, and intestinal microbiota generally cannot enter other organs under healthy conditions. An article on HFD-fed mice may provide a mechanism for intestinal vascular barrier leakage and the passage of macromolecules and bacteria, due to the translocation of intestinal bacteria transferred to the liver and the destruction of intestinal vascular barrier by bacteria or virulent factors (42). However, with the help of the pathogenicity island 2-encoded type III secretion system and reduced intestinal endothelial cell-dependent β-catenin signaling, certain pathogens can penetrate the intestinal vascular barrier to reach these organs and induce systemic immune response (43). The leakage of bacteria and their metabolites can also affect the function of the vascular wall barrier of the brain (44, 45), eyes (46), and testes (18). Our results indicate that obesity leads to reduced sperm motility, affects sperm quality, destroys the BTB, and causes a highly inflammatory state in zebrafish testis. Endotoxins can dramatically increase the permeability of the intestinal wall and damage the mucosa to form inflammation and ulcers (47, 48). Therefore, we speculate that the disturbances of intestinal microbes may affect testicular microbes through the leakage of pathogenic bacteria and their metabolites.

A study on intestinal microbes and sperm quality indicated that endotoxemia and epididymal inflammation are caused by an imbalance in intestinal microbiota in mice, which are the main factors attributed to sperm quality and motility. The authors transplanted intestinal microorganisms from HFD-fed mice to mice fed with a normal diet. After 15 weeks, the endotoxins in the blood nearly increased by threefold, and sperm motility was also affected (49). The results suggested that obesity alters intestinal microbiota composition and reduces sperm motility. This finding further validated the speculation that intestinal microorganisms may be transferred to the testis *via* the destruction of the intestinal vascular barrier and through the blood, thereby affecting testicular microbiota composition.

A study on the intestinal microbiota of individuals with polycystic ovary syndrome (PCOS) and clinical indicators associated with imbalanced microbiota found substantial differences in the types of intestinal microorganisms between PCOS and non-obese control groups. A positive correlation was observed between the abundance of *Shigella* and *Streptococcus* with testosterone and BMI (50). PCOS is a systemic disease of the female genital ovaries related to obesity (51). In male reproductive diseases associated with obesity, *Escherichia-Shigella* may be positively correlated with testosterone and BMI.

## Conclusion

We speculate that the disturbance of intestinal microbes causes the imbalance of testicular microbiota through the production of endotoxemia, which increases the richness of *Escherichia-Shigella* and causes testicular inflammation. On the one hand, *Escherichia-Shigella* promotes the process of endotoxemia, and on the other hand, it further exacerbates testicular inflammation, leading to orchitis. Under the action of a large number of inflammatory factors and toxins, the BTB is damaged. While affecting the structure of the testis, the physiological functions of the testis were also adversely affected. Testosterone levels and sperm quality dramatically decreased. In short, the decline of sperm motility in obesity may be caused by an imbalance in testicular microbiota, leading to the destruction of the BTB structure and inflammatory response in the testis. Despite our findings, this study has some limitations. For example, our sample size is limited. And because of the small size of zebrafish (the mean testis weight of zebrafish was 4.7 ± 0.2 mg), the microbiological samples we collect are mixed samples.

## Data Availability Statement

The datasets presented in this study can be found in online repositories. The names of the repository/repositories and accession number(s) can be found below: NCBI AND PRJNA722878.

## Ethics Statement

The study was conducted in strict accordance with the guidelines approved by the Animal Care and Use Committee of Tongji Medical College, Huazhong University of Science and Technology.

## Author Contributions

YS and CL designed research studies, conducted experiments, analyzed data, and drafted the manuscript. ZH assisted in 16sRNA mapping analysis. LH and YZ conducted Zebrafish model establishment. KZ and YL performed zebrafish execution and electron microscopy. ZF conducted sample collection and storage. CL and HZ provided intellectual input into planning of experiments and contributed to the writing of the manuscript. All authors contributed to the article and approved the submitted version.

## Funding

This study was supported by National Key Research and Development Project (2018YFC1004300) and Wuhan Youth Science and technology plan (2017050304010291).

## Conflict of Interest

The authors declare that the research was conducted in the absence of any commercial or financial relationships that could be construed as a potential conflict of interest.

## Supplementary Material

Supplementary Figure 1 Analysis of the microbial community composition of the intestinal samples on the phylum level.

Supplementary Figure 2 Analysis of the microbial community composition of the testis samples on the phylum level.

Supplementary Figure 3 Analysis of the relationship between samples and species of two groups of testicular microorganisms was performed to show the distribution ratio of dominant species in each group of bacteria and the distribution ratio of each dominant species in different groups.

Supplementary Figure 4 Species composition at different levels of the testicular bacterial community at the gate level and heatmap and sample cluster tree analyses.

Supplementary Figure 5 Venn plot population analysis results of all samples, including gut and testicular microbes.
