# Supplementary figures and images for: Obesity Causes Abrupt Changes in the Testicular Microbiota and Sperm Motility of Zebrafish
Source: Front Immunol. 2021 Jun 25;12:639239. doi: 10.3389/fimmu.2021.639239 (PMC8268156; doi:10.3389/fimmu.2021.639239)

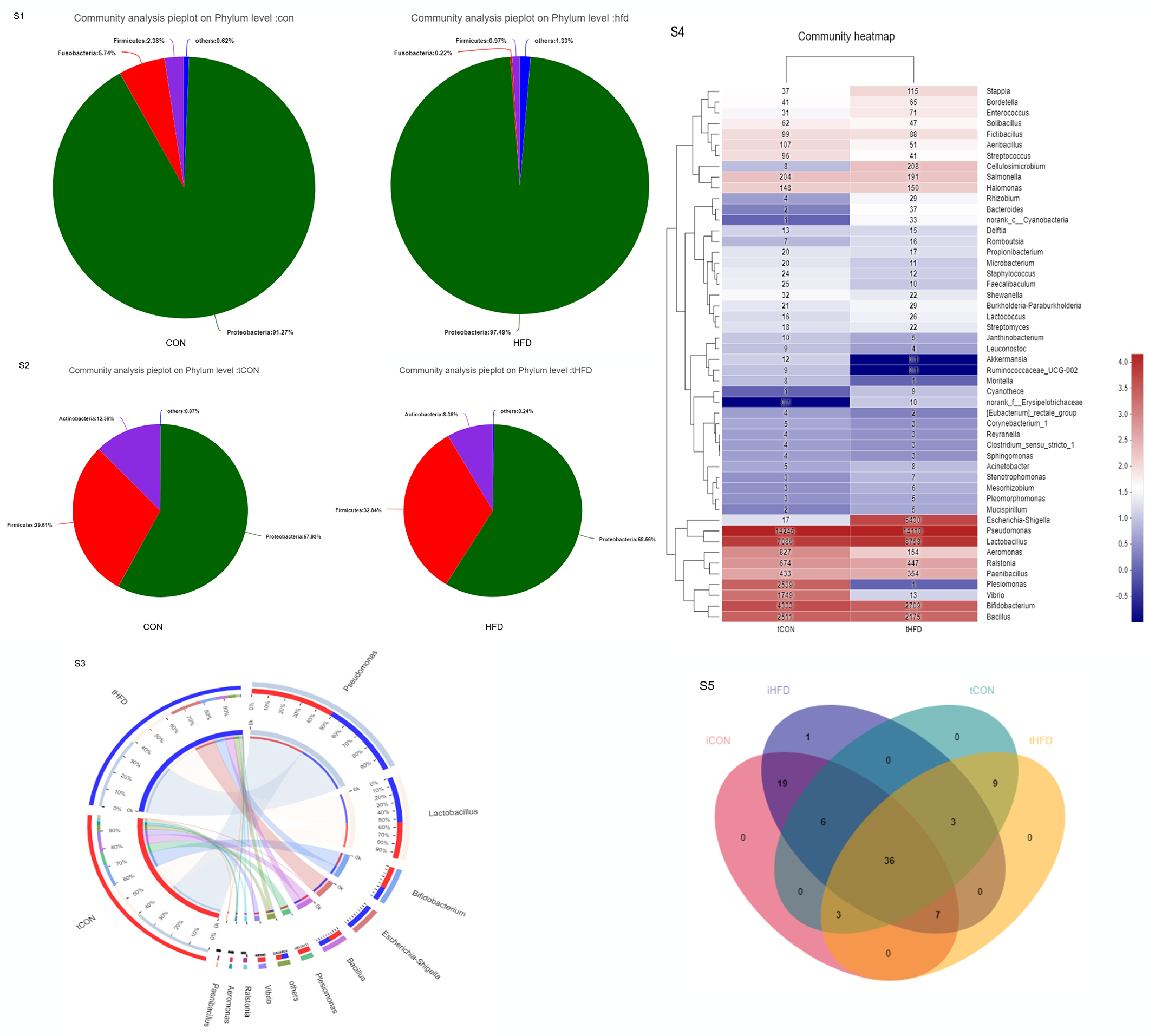

Supplement: Supplementary Figure 1 — Analysis of the microbial community composition of the intestinal samples on the phylum level. [file Image_1.tif]
